# Supplementary material for: Genome-Wide Association Study of Muscle Glycogen in Jingxing Yellow Chicken
Source: Genes (Basel). 2020 Apr 30;11(5):497. doi: 10.3390/genes11050497 (PMC7290304; doi:10.3390/genes11050497)
Supplement: Supplementary file 1 [file genes-11-00497-s001.pdf]

genes

Supplementary materials

Table S1. SNP and INDEL corresponding serial number.

NAME	Chromosome	Site
SNP1	1	150449184
SNP2	2	42074647
SNP3	2	42078810
SNP4	3	26983490
SNP5	3	101450476
SNP6	4	15746019
SNP7	4	18209706
SNP8	4	25507719
SNP9	11	6224928
INDEL1	1	7169549
INDEL2	3	27425548
INDEL3	11	6376492

Figure S1. Potentially related phenotypic differences among 9 SNPs and 3 INDELs with different genotypes. (**a**–**l**) The abscissa SNP 1–9 and INDEL 1–3 is the genotype of potential related sites, followed by wild type, heterozygous type, and mutant type. The ordinate Glycogen is the muscle glycogen content

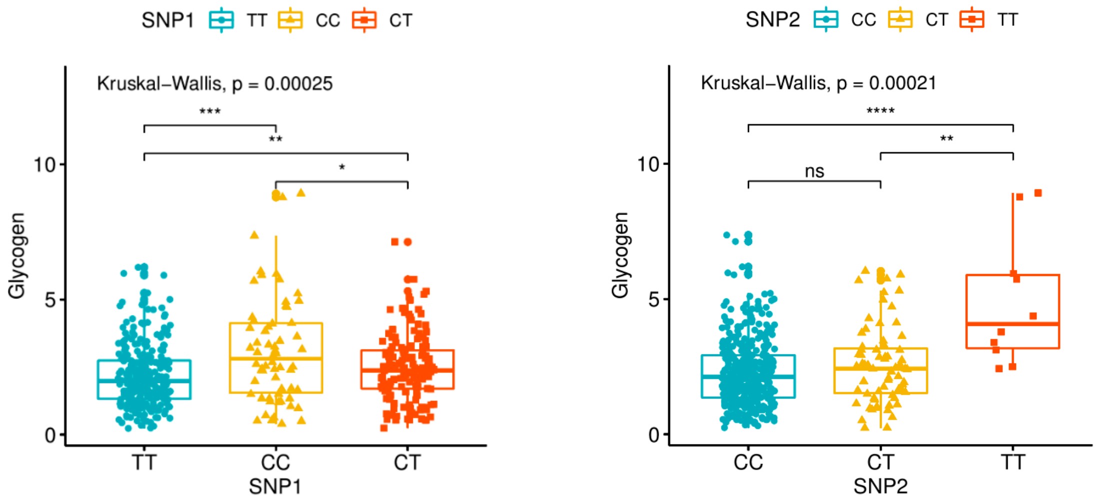

(**a**) (**b**)

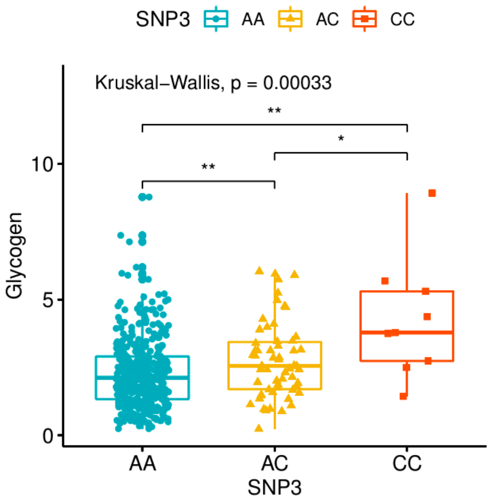
SNP3 AA AC CC
Kruskal–Wallis, p = 0.00033
Glycogen
AA AC CC
SNP3

(**c**)

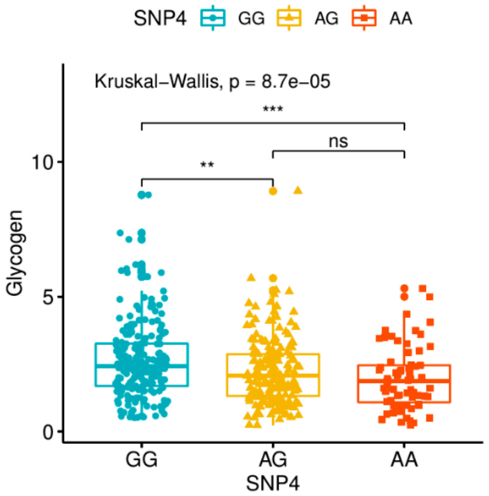
SNP4 GG AG AA
Kruskal–Wallis, p = 8.7e–05
ns
Glycogen
GG AG AA
SNP4

(**d**)

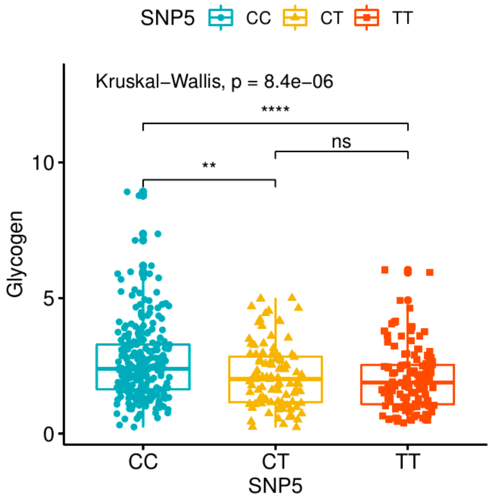
SNP5 CC CT TT
Kruskal–Wallis, p = 8.4e–06
ns
Glycogen
CC CT TT
SNP5

(**e**)

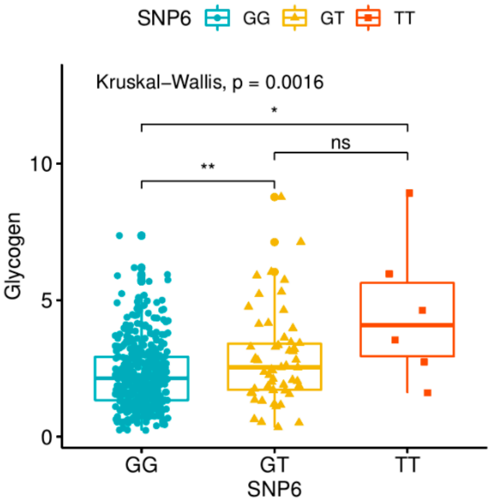
SNP6 GG GT TT
Kruskal–Wallis, p = 0.0016
ns
Glycogen
GG GT TT
SNP6

(**f**)

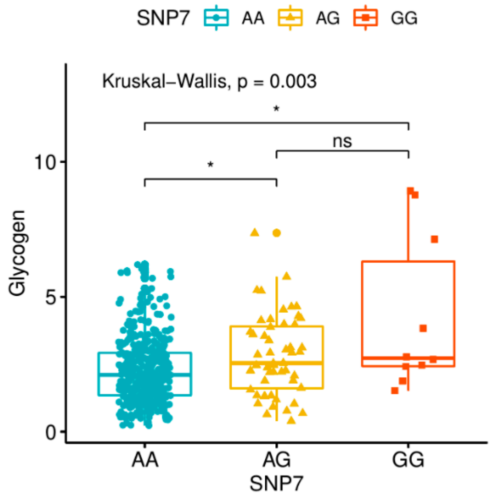
SNP7 AA AG GG
Kruskal–Wallis, p = 0.003
ns
Glycogen
AA AG GG
SNP7

(**g**)

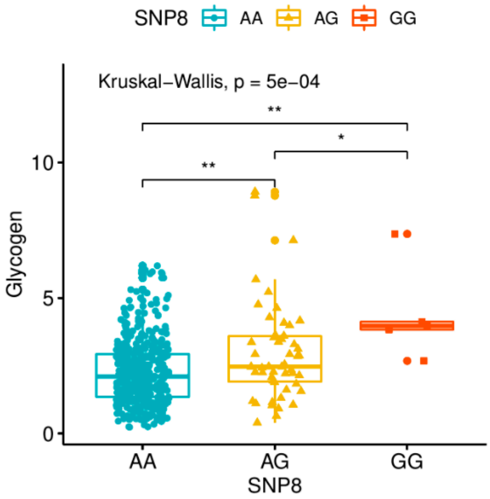
SNP8 AA AG GG
Kruskal–Wallis, p = 5e–04
Glycogen
AA AG GG
SNP8

(**h**)

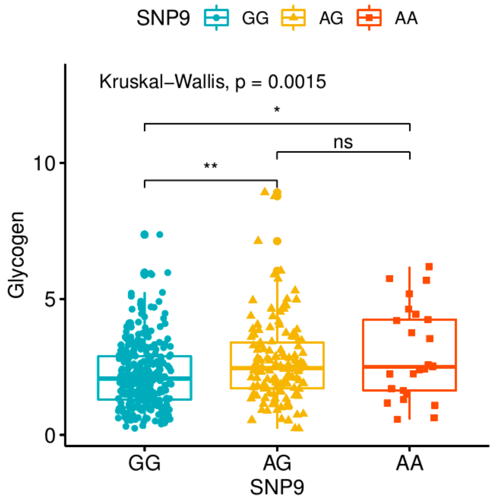
SNP9 GG AG AA
Kruskal–Wallis, p = 0.0015
*
ns
**
Glycogen
0
5
10
GG
AG
AA
SNP9

(**i**)

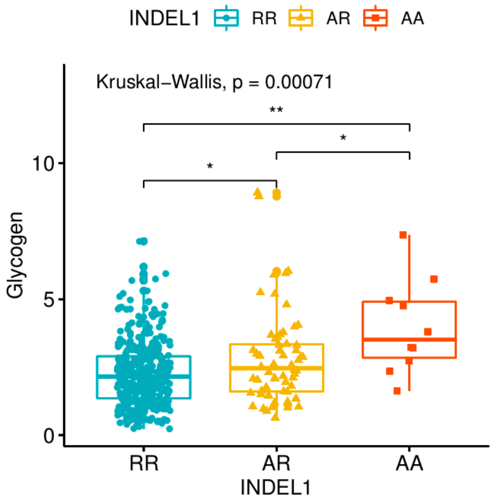
INDEL1 RR AR AA
Kruskal–Wallis, p = 0.00071
**
*
*
Glycogen
0
5
10
RR
AR
AA
INDEL1

(**j**)

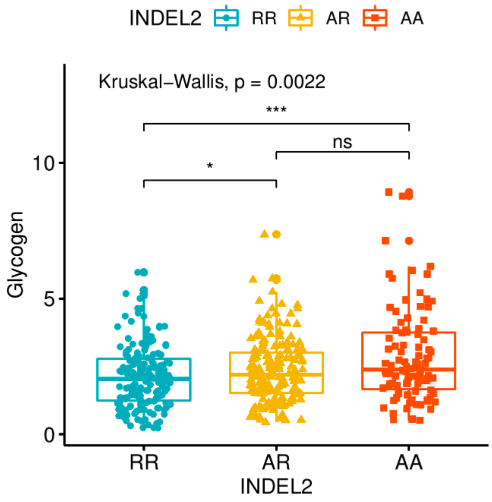
INDEL2 RR AR AA
Kruskal–Wallis, p = 0.0022
***
ns
*
Glycogen
0
5
10
RR
AR
AA
INDEL2

(**k**)

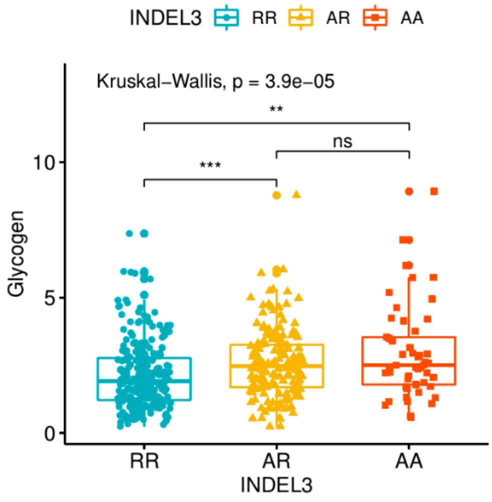
INDEL3 RR AR AA
Kruskal–Wallis, p = 3.9e–05
**
ns
***
Glycogen
0
5
10
RR
AR
AA
INDEL3

(**l**)
